# CHILDHOOD TEMPERAMENT-BASED ANTICIPATORY GUIDANCE IN AN HMO SETTING: A LONGITUDINAL STUDY

**DOI:** 10.1002/jcop.21526

**Published:** 2013-02-14

**Authors:** James R Cameron, David C Rice, Gregg Sparkman, Helen F Neville

**Affiliations:** The Preventive Ounce; Kaiser Permanente

## Abstract

This study investigates whether individualized, anticipatory temperament guidance could benefit the parent-child relationship and improve children's mental health over time. Parents of preschoolers in a health management organization completed a temperament questionnaire, received written parenting information tailored to their child's temperament, and were asked to complete a program evaluation questionnaire. The numbers of subsequent visits to the pediatric and psychiatry departments with anxiety, depression, attention deficit hyperactivity disorder, and other externalizing behavior diagnoses were compared over 15 years to a control sample that received only standard care. Parents positively reviewed the program and boys who received the intervention had fewer visits with psychiatric diagnoses. Analyses revealed an interaction effect, where boys with harder-to-manage temperaments saw a greater reduction in visits from the intervention. By sensitizing parents to their child's temperament and helping parents understand and manage temperament-related behaviors, anticipatory guidance can encourage a positive parent-child relationship and reduce future occurrences of psychiatric diagnoses.

Kaminski, Valle, Filene, and Boyle (2008), in a meta-analysis of 128 parent training programs, evaluated a variety of content, delivery settings, and delivery techniques that targeted a variety of parents who were either experiencing behavioral problems with their children or at risk of experiencing behavioral problems. They asked what components of these parent training programs were most effective in helping parents be more successful with their children and found that the “program components consistently associated with larger effects included increasing positive parent-child interactions and emotional communication skills” (p. 567) as well as teaching specific parenting tools and having parents practice during class what they were learning. To increase positive parent-child interactions, particularly if the goal is to develop prevention programs, it is helpful to understand the mechanisms of how childhood problems develop (Frick & Morris, 2004). Research is also needed to clarify what mediators and moderators influence parent-child interactions when children develop childhood disorders (Eyberg, Nelson, & Boggs, 2008; Silverman & Hinshaw, 2008).

Unfortunately, large-scale intervention programs designed to improve positive parent-child interactions would be prohibitively expensive if they used professionals for in-person assessment and intervention. However, intervening from a childhood temperament perspective offers a possible solution to this problem, because childhood temperament can be measured with parental questionnaires and parents can receive computer-generated feedback about how to respond more effectively to their child's specific temperament-related behaviors. In that regard, the present study examines the utility and outcomes of a written temperament-based anticipatory guidance program for parents.

Four decades of evidence, including two longitudinal studies, document a robust relationship between the innate behavioral patterns of childhood temperament, parenting, and subsequent childhood behavioral problems (Cameron, 1978; Guerin, Gottfried, Oliver, & Thomas, 2003; Laucht, Schmidt, & Esser, 2008; Prior, Sanson, Smart, & Oberklaid, 2000; Thomas, Chess, & Birch, 1968). In addition, research shows that childhood temperament and parenting influence each other, both as distinct influences and through interactive effects on a child's behavior (Putnam, Sanson, & Rothbart, 2002). Therefore, if parents have a clearer understanding of their child's temperament, the parent-child relationship and the child's well-being should improve.

More recent research has linked temperament to specific childhood psychological diagnoses. This includes the externalizing diagnoses of attention deficit hyperactive disorder (ADHD; Bussing, Lehninger, & Eyberg, 2006) and oppositional defiant disorder or conduct disorder (Campbell, Shaw, & Gilliom, 2000; Rettew, Copeland, Stanger, & Hudziak, 2004), as well as the internalizing diagnoses of anxiety (Goodwin, Fergusson, & Horwood, 2004; Inca, Benga, & Fox, 2006) and depression (Pitzer, Jennen-Steinmetz, Esser, Schmidt, & Laucht, 2011). Though this is known, Muris and Ollendick (2005) have argued that “research on … the predictive value of temperament on the development of psychological problems [is] urgently needed” (p. 284). Frick (2004) and Shiner and Caspi (2003) have pointed to the lack of research integrating temperament and childhood psychopathology and the importance of understanding the different ways temperament influences psychopathology, to develop more effective treatment programs for children.

When measuring temperament with questionnaires, parent and observer ratings do not necessarily agree (Stifter, Willoughby, & Towe-Goodman, 2008). While this has led some to question the validity of parent ratings, “it would be more realistic to take a components of variance approach. Parents, as well as observers, vary in the degree to which subjective and objective factors contribute to their ratings” (Stifter et al., p. 423). Rothbart and Bates (1998) concluded that parent ratings “provide a useful perspective on the personality of children … and … have established a fair degree of objective validity” (p. 126). Given that parent perceptions of childhood temperament affect parent-child interactions (Rothbart & Bates, 1998), parents’ ratings of their child's temperament have clinical value in a prevention program.

Beginning in 1979, we conducted a series of studies to investigate if providing anticipatory guidance to parents could improve the parent-child relationship beyond the efforts of normal pediatric care, which may not include advice based on the child's temperament. We generated advice for parents based on the nine temperament scales developed by Chess and Thomas (Thomas, Chess, & Birch, 1968). We anticipated that guidance tailored to each child's unique temperament could help parents understand and cope with temperament-related behaviors better than either general parenting advice or generalized information about temperament.

In a previous study we found that parents reported a greater understanding of temperament-related behaviors when they received anticipatory guidance. This advice was also rated as more useful by parents who reported a higher incidence of problem behaviors (Cameron & Rice, 1986). However, questions still remained whether such guidance would actually benefit children, by either improving the parent-child relationship or reducing the occurrence of problematic temperament-related behaviors. To further evaluate the feasibility of providing effective temperament-related anticipatory guidance, we conducted the present study and included (a) more detailed parent evaluation measures and (b) the frequency of children's visits with internalizing and externalizing diagnoses to a large health maintenance organization (HMO).

We expected effective anticipatory guidance would improve the parent-child relationship by making parents sensitive to temperament and to temperament's influence on their child's behavior. Therefore, our first hypothesis is that the parents in the intervention group will report the guidance helped them see their child more clearly, understand how temperament contributes to their child's behavior, and change their expectations of what is normal for their child's behavior. We also expected intervention parents would rate the support positively and recommend the program to others.

Given an improvement in the parent-child relationship, we expected intervention parents’ children would have a decrease in visits to their health maintenance organization (HMO) associated with temperament-related behaviors, either because the guidance helped parents more effectively manage those behaviors or because an improved parent-child relationship led to fewer temperament-related behavioral problems. However, a decrease in visits to an HMO should not be due to parent's becoming discouraged from seeking needed treatments for their children. Therefore, our second hypothesis is that children in the intervention group will have fewer behavior-related visits than the control group, and the intervention group parents will report no changes in the amount of advice sought from their HMO compared with the control.

Parents who report children with harder-to-manage temperaments are more likely to have trouble understanding and managing their child (Crockenberg, 2008) and may think there is something wrong with their child or their parenting. We anticipated that our individualized guidance would have a greater effect for these parents. Therefore, our third hypothesis is that the effectiveness of the intervention will be greater for children with harder-to-manage temperaments.

## METHODS

### Participants

Parents and children were members of Kaiser Permanente of Northern California who were receiving services at the Oakland, San Rafael, Santa Rosa, Sacramento, and Vallejo facilities between 1992 and 2007. Girls accounted for 46.3% of the sample. Demographic information on race, ethnicity, educational level, and socioeconomic status was not collected. No children were excluded from the sample. Of the facilities where participants were recruited, Oakland, Sacramento, and Vallejo are more racially, ethnically, and financially diverse than San Rafael and Santa Rosa, which are largely Caucasian and middle or upper-middle class. Parents could bring children to any facility for care as a Kaiser Permanente member, and we do not know which facility was the primary facility for each child.

The study was approved by the Kaiser Permanente Institutional Review Board. All parents of 3.5-year-old preschoolers at the study facilities during the study period were sent a temperament questionnaire and an invitation by mail to join the study. Of the parent's contacted, 662, or 18.4%, returned the questionnaire. Of these respondents, 55 were removed from the present study for having incomplete questionnaire data and are excluded from all future analysis. There were no significant differences between the intervention (N = 327) and control (N = 280) response rates c^2^(1, *N* = 607) = 1.00, *p* = .3176. All parents who returned the temperament questionnaire were sent a study evaluation questionnaire when their child was 5 years old. Of those parents, 366, or 60.2%, returned the study evaluation with no significant differences between the intervention (N = 187) and control (N = 179) groups, c^2^(1, *N* = 607) = 2.06, *p* = .151 Of the 607 respondents, 458 (75.3%) maintained their membership at the HMO through the course of the study. There was no significant difference between control (N = 216) and intervention (N = 242) group attrition rates, c^2^(1, *N* = 458) = 0.77, *p* = .390.

### Measures

At study entry, intervention and control group parents filled out a preschool Cameron-Rice temperament questionnaire (Cameron & Rice, 1989) based on the nine temperament dimensions (activity, intensity, approach/withdrawal, adaptability, distractibility, sensitivity, regularity, persistence, and mood) initially defined by Chess and Thomas (Thomas, Chess, & Birch, 1968) and later measured in preschoolers by McDevitt and Carey (1978). The Cameron-Rice questionnaire used a 6-point Likert scale and modified these previous questionnaires by writing items that pertained more directly to the guidance and eliminating items that did not pertain to the guidance.

To measure the utility of the guidance, intervention parents filled out a 5-item follow-up questionnaire, using a 2-, 3- or 5-point ordinal scale, about how well the guidance helped them see and understand their child's temperament-related behavior. Both intervention and control group parents were asked how the study affected their utilization of medical services for behavioral issues.

To measure utilization of medical services for behavioral issues, we examined visit-data from the HMO. When parents took their child to a Kaiser Permanente clinic, the clinic routinely recorded the reason for the appointment by using an International Classification of Diseases, Ninth Revision (ICD-9) diagnostic code. The diagnosis reflected the health care provider's assessment of the reason for the child's visit; it may or may not have reflected the reason the parents brought the child to the clinic. We obtained the date and frequency of the childhood diagnoses of externalizing behavior (ADHD/ADD and other externalizing behaviors) and internalizing behavior (anxiety and depression). Table 1 shows the frequency of each diagnosis.

**Table 1:** Percentage of Studied Visits for Total Sample by Diagnostic Category

	Boys visits	Girls visits	Total visits
Diagnosis	(N = 1303)	(N = 1928)	(N = 3231)
ADHD/ADD	30.62%	7.94%	17.08%
Anxiety	15.58%	18.57%	17.36%
Depression	19.19%	40.09%	31.66%
Other externalizing behaviors	34.61%	33.40%	33.89%

ADHD = attention deficit hyperactive disorder.

High or low scores on certain Chess and Thomas temperament scales are more likely to be harder to manage for parents (Chess & Thomas, 1977). For hypothesis 3, we created a harder-to-manage temperament composite score that gave more weight to scores for high activity, high intensity, low persistence, low regularity, slow adaptability, and negative mood, as well as either high or low scores for sensitivity, distractibility, and approach/withdrawal.

### Procedure

From 1992-1994, using the HMO's list of members who were 3.5 years of age each month, we alternately assigned each child to the intervention or control group. An invitation letter informed both intervention and control parents that if they returned the enclosed questionnaire, a copy of their child's temperament profile would be placed in their child's medical record. Parents invited to the intervention group were also informed that if they participated, they would receive a profile of their child's temperament and written guidance that would include (a) general information emphasizing that variations in temperament were normal, (b) a discussion of the behavioral issues likely to occur for their child's specific temperament, and (d) management strategies for these temperament-related behavioral issues. Eighteen months after receiving the guidance, when the child reached 5 years of age (from 1993 to 1995), parents were sent a guidance evaluation questionnaire. Parents were not contacted during the remainder of the study years or thereafter.

We collected the ICD-9 diagnostic codes for all visits between 1995 and 2008. Because Kaiser Permanente did not computerize its record system until 1995, we were unable to obtain patient utilization of medical services data prior to 1995.

### Intervention

Intervention parents received written guidance by mail for behaviors expected to occur over the following 18 months in six areas that had previously been found to be temperament-related: accident risk, assertiveness, mealtime, sensitivity, separation-dependency, and sleep (Cameron & Rice, 1986), as well as assertiveness/response to direction and limits, daycare/preschool, and new situations.

Our previous studies and clinical experience indicated which temperament scales were associated with particular temperament-related behaviors. To generate the specific advice for each child, we assigned a low, moderate, or high likelihood of occurrence of harder-to-manage behaviors for each area, based on the temperament profile. Parents were mailed a packet of information on parenting and temperament that included the following: (a) an introductory letter stating that the purpose of the program was to help parents see their child more clearly and that variations in temperament are normal; (b) the child's temperament profile, showing the child's score on each of the nine temperament scales, and information on how to read the profile; (c) a two-page description of how individual high or low temperament scores, and combinations of temperament scores, were likely to affect day-to-day behavior (this section also indicated that such behaviors were normal for a child of that temperament and included general management strategies for that temperament profile); and (d) up to two pages of advice for each specific behavioral issue associated with this child's temperament profile, which explained how to manage those behaviors in ways that were appropriate for the child's temperament. Parents of children who were not likely to have a temperament-related issue in a behavioral area were told why their child's temperament meant these issues would probably not occur. The senior author wrote this material.

For example, a slow-adjusting and moderate-energy preschooler advice sheet on daycare/preschool read in part:

Adjusting to preschool can take a little time for preschoolers like yours, who are moderate in their energy level and who adjust more slowly in new or strange situations… The first few days of preschool (or day care) your child may show signs of “separation anxiety.”… Once inside the preschool, his first reaction is likely to be to stick to the sidelines, watching and learning. Unlike the more active children, his energy level won't propel him into the school's activities… As the weeks go by, parents hear reports of how assertive and independent he is becoming. But once their child returns home, parents find he is now more dependent, demanding their attention and seeking out their support. The reason is that each school day, they exhaust their adaptability in coping with the demands of school. Once they escape those school expectations, their dependent side is released. Parents naturally ask, “How should I respond…?” Some structure for the late afternoon will help… a quiet walk outside, perhaps a short trip to a nearby park… can allow him to organize and release the rest of his energy and gradually slow down for dinner and bed.

### Statistical Analysis

We conducted separate analyses for each gender, because there are gender differences in childhood temperament (Else-Quest, Hyde, Goldsmith, & Van Hulle, 2006) and in childhood psychopathology (Hankin & Abramson, 2001; American Psychiatric Association, 1994).

To evaluate the effectiveness of the parental guidance for hypothesis 1, we report the descriptive statistics for the intervention group's follow up survey. To evaluate the effect of the intervention for hypothesis 2, we calculated independent sample *t* tests, Cohen's d, and effect sizes to compare differences in visits between the control and intervention groups for boys and girls. We repeated these tests after removing outliers past the fourth standard deviation. To explore the significant differences found in testing hypothesis 2, we further analyzed the results by dividing the visits by participants’ age and by diagnosis.

To test whether the intervention had a significantly different effect on easier or harder-to-manage temperament for hypothesis 3, we ran a moderated multiple regression model (Jaccard, Guilamo-Ramos, Johansson, & Bouris, 2006) using a mean centered hard-to-manage temperament composite score, a dichotomous intervention variable, and a temperament-intervention interaction term. The R^2^ value was compared with and without the interaction term in the model.

## RESULTS

### Hypothesis 1: Intervention Parent Feedback Will Be Positive

When reporting if they saw their child more clearly because of the intervention, 40.2% of intervention parents said “Yes, a lot more,” 52.5% said “Somewhat,” and 7.4% said “No, not at all.” When reporting if the intervention helped them understand how temperament contributed to their child's behavior, 49.2% said “Yes,” 42.6% said “Somewhat,” and 8.2% said “No.” When reporting if the intervention changed their expectations of what was “normal” for their child, 50.0% said “Yes,” 27.9% said “Somewhat,” and 22.1% said “No.” When asked to rate the support they received from the intervention, 20.9% said “Excellent,” 50.9% said “Good,” 20.9% said “Fair,” 0% said “Poor,” and 7.3% said “There was no support.” When asked if they would recommend the intervention to other parents, 97.5% said “Yes” and 2.5% said “No.”

### Hypothesis 2: Intervention Children Will Have Fewer Behavior-Related Visits

The intervention had a significant effect on boys’ visits, where the intervention group had fewer visits than the control, as shown in Table 2. There were no male outliers past the fourth standard deviation in visit frequency. There was no significant effect on girls’ visits, with or without outliers.

**Table 2:** Intervention Effect on Boys’ Psychiatry Visits Over 15 Years

N	Control Mean(SD)	Intervention Mean(SD)	T	Cohen's *d*	*r* effect size
208	7.91(16.50)	4.58(10.18)	1.75*	.243	.121

SD = standard deviation.

* *p* < .05.

When asked if the intervention led parents to increase or decrease the amount of advice they sought from their HMO regarding their child, 20.8% of intervention parents selected “Increase,” 11.7% said “Decrease” and 67.5% said “No change.” There was no significant difference between the intervention or control groups’ responses, F(1, 224) = 1.286, p = 0.258.

To see when the difference in boys’ visits occurred, we repeated this analysis after dividing visits by child's age into 3-year periods, and found the visit reduction primarily between 4 and 10 years of age, as shown in Table 3.

**Table 2:** Intervention Effects on Boys’ Psychiatry Visits by Age (N = 208)

Age	Control Mean(SD)	Intervention Mean(SD)	T
4–7	2.43(5.27)	0.58(1.77)	2.50**
7–10	2.28(6.39)	0.88(2.42)	1.70*
10–13	2.76(6.51)	1.76(4.38)	1.12
13–16	3.79(8.08)	2.64(6.10)	0.97
16–19	1.84(5.80)	1.03(2.42)	0.80

SD = standard deviation.

* *p* < .05.

** *p* < .01.

When we separated boys’ visits by diagnosis, the intervention group had significantly fewer visits for anxiety (p < .005), compared with the control group, and a near significant reduction for externalizing behavior diagnoses other than ADHD (p = .069), as shown in Table 4.

**Table 4:** Intervention Effects on Frequency of Boys’ Psychiatric Diagnoses (N = 208)

Diagnosis	Control Mean(SD)	Intervention Mean(SD)	T
ADD/ADHD	2.27(6.82)	1.56(4.12)	0.90
Anxiety	1.57(4.29)	0.37(1.23)	2.74**
Depression	1.33(4.48)	1.07(4.33)	0.43
Other Externalizing behaviors	2.74(6.81)	1.58(4.01)	1.49†

*Note*: SD = standard deviation.

† *p* < .10,

** *p* < .005.

### Hypothesis 3: The intervention Effect Will Be Greater for Children With Harder-to-Manage Temperaments

**Figure 1:**
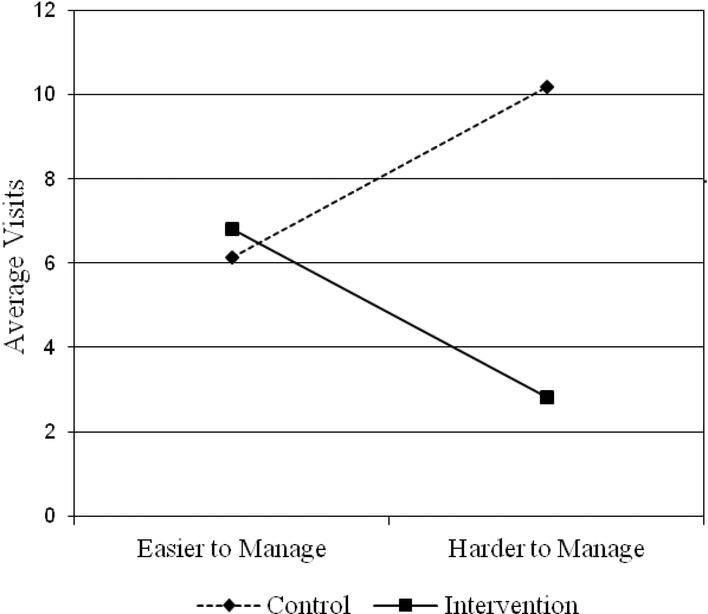
Reduction in visits from intervention increases with harder-to-manage temperament.

To look for a moderation of harder-to-manage temperaments on the effectiveness of the intervention, we conducted a multiple regression using the intervention condition, temperament scores, and the interaction term as outlined in the methods section. In this model, belonging to the intervention group marginally predicted fewer visits, β = −.130, t(208) = −1.88, *p* = .062, harder-to-manage temperament did not predict fewer visits, β = .152, t(208) = 1.48, *p* = .139, and the interaction term significantly predicted visits, β = .219, t(208) = 2.151, *p* = .033. The model, F(1, 206) = 2.582, *p* = .055, saw an increase in R^2^ of .022, from .015 to .037. In the interaction, the reduction in visits from the intervention was greater for those with harder-to-manage temperaments, as seen in Figure 1.

## DISCUSSION

Parents rated the intervention positively. More than 90% of intervention parents said the guidance helped them see their child a lot more clearly or somewhat more clearly as well as better understand how temperament contributed to their child's behavior. More than 75% said the guidance changed their expectations of what was “normal” for their child. More than 70% rated the guidance as excellent or good support and more than 95% would recommend the program to other parents. These results support hypothesis 1 and suggest that the intervention helped parents become more sensitive to their child's temperament and its relationship to their child's behavior.

For boys, the intervention group averaged about three fewer visits with a psychiatric diagnosis over the course of the study; this decrease occurred primarily when the boys were between 4 and 10 years of age. Compared with the control group, the intervention group parents reported no significant difference in whether the program changed the amount of advice sought from the HMO. This suggests the guidance did not lead intervention parents to neglect actual problems and avoid treatment when treatment was warranted, nor become overly concerned and seek treatment when treatment was unwarranted. Children also are routinely seen by pediatricians, who can make referrals to the psychiatry department if they believe the child should receive psychological treatment. It is most likely, then, that, by improving their understanding of their child's temperament, intervention parents were able to more effectively manage temperament-related behaviors or experienced fewer temperament-related problems as a result of the intervention, supporting our second hypothesis. For boys with harder-to-manage temperaments, where there is a greater risk to a positive parent-child relationship, the intervention's effect was greater. This finding supports our third hypothesis and suggests a temperament-based parent-training program can be effective for boys with harder-to-manage temperaments.

While our intervention group saw a significant difference in the average number of psychiatric diagnoses (roughly a 43% reduction), and had a greater effect on children with harder-to-manage temperaments, the large variability of visit frequency led to a small effect size for our intervention. This variability is expected, since we used a general sample that included both clinical and nonclinical children. More generally, the relationship between psychopathology and temperament occurs over time and has a broad spectrum of intervening variables that influences childhood behavior, including the interaction of temperament and parenting, all of which produces great variability and decreases the likelihood of finding large effect sizes (Shiner & Caspi, 2003). Given the complex relationship of temperament and psychopathology, “even associations with small effect sizes can be of theoretical and practical significance” (Muris & Ollendick, 2005, p. 284).

The outcomes of this study suggest that long-term improvements to the parent-child relationship are possible with this form of intervention. In part, our guidance contained time-sensitive advice on specific behaviors, and we expected that this guidance would be most effective in the years immediately after the parents received it. However, the more general information about how their child's temperament affects day-to-day behavior, and the tailored management strategies, continued to be relevant throughout adolescence. Given the time frame of the visits measured, the present study investigates the long-term effect of temperament guidance on the parent-child relationship, rather than the prevention of the specific, time-sensitive temperament-related behaviors discussed in the intervention. One example of a long-term outcome from this intervention was the reduction of boys’ anxiety diagnoses. This also supports previous work that has found parent training programs are more effective with internalizing diagnoses (Kaminski et al., 2008).

An anecdote from a Kaiser Permanente psychologist provides one explanation of how the advice may have led to a decrease in behavioral visits in the intervention group. She observed that when parents who had not received temperament-related anticipatory guidance brought their child to the clinic due to ADHD, she had to both treat ADHD and try to repair the parent-child relationship. When parents who had received the guidance brought their child to the clinic, she only had to treat the ADHD. This further suggests if parents have a child with a harder-to-manage temperament, they are at risk of getting off to a poor start in managing their child's temperament-related behavior. Over time, this may harm the parent-child relationship and later require more intensive intervention.

### Limitations

A major limitation of the study, resulting from confidentiality and budget constraints, is the absence of an alternative measure of how well the children were functioning, independent of the parent's evaluation of the guidance and the frequency of visits sought for behavioral-related issues. While the present study indicates it is likely that the guidance helped parents of boys improve their relationship with their children, benefiting the children and leading to fewer behavior-related visits, we do not have confirmatory evidence from school records, family assessments, or other direct measures of the children's functioning.

Sample limitations also exist. Demographic information was not collected and the response rate was relatively low. While the response rate may be due to sending only a single invitation to parents, it is also possible the participants do not constitute a representative sample. Past research has shown that participants of higher socioeconomic status are more likely to respond to questionnaires (Heberlein & Baumgartner, 1978). Parents who did not join the study may not have been comfortable sending and receiving written information due to their educational level or because English was not their first language. Without demographic information, it is impossible to know whether our sample included primarily higher socioeconomic parents or if socioeconomic status moderated the utility of the guidance. It is also possible that other self-selection factors, such as a greater response from parents who were particularly struggling with their child's behavior, or, alternatively, from parents who spend a greater than average amount of time seeking out parenting resources, may have biased the sample. We lack information on whether parents sought psychological services outside of their HMO coverage. Also, missing data from 1992 through 1994, the years immediately after the intervention, may have affected the results.

Temperament assessment has improved in the past 25 years. Contributions from contemporary work, which have measured broad temperament factors such as effortful control, surgency, and negative affectivity (Putnam, Ellis, & Rothbart, 2001) as well as specific temperament scales, are likely to produce a more valid and predictive assessment of childhood temperament than the nine Chess and Thomas scales used in the present study. Improved temperament assessment, along with advice generated from combinations of contemporary temperament scales, are likely to produce more effective guidance. Incorporating contemporary material on temperament into the intervention could generate significant results for a broader population, including girls.

### Implications

Putnam, Sanson, and Rothbart (2002) distinguish between the view that temperament affects parenting and the view that parenting affects temperament. If temperament affects parenting, then interventions need to focus on helping parents be more aware of their child's temperament and avoid ineffective parenting reactions. If parenting affects temperament, then interventions need to focus on helping parents help their child learn to manage temperament-related issues. Our study focused on helping parents become aware of their child's temperament. We found that parent questionnaires and targeted behavior-management recommendations can familiarize parents with temperament-related behaviors that are likely to occur and are “normal” for their child, as well as reduce behavior-related HMO visits. Given the limitations of the present study, replication with current methodological standards and relevant control variables is necessary before the true benefit of this type of intervention can be assessed. However, these initial results suggest that children can benefit from an intervention that likely improves parent's sensitivity and ability to integrate temperament awareness into their parenting methods.

Kaiser Permanente's Northern California and Oregon facilities currently utilize a second-generation online version of this intervention. If web-based, childhood temperament anticipatory guidance can be shown to improve the parent-child relationship and directly benefit children across a socioeconomically diverse range of families, then it would be feasible to offer large-scale preventive programs to reduce future temperament-related childhood behavioral problems. Such a program, augmented with data about parent variables, also would have the potential to reveal some of the mediators and moderators of childhood behavioral disorders, since it is the interaction between childhood temperament and parenting that best predicts potential childhood behavioral problems. Given the growing awareness of the benefits of prevention over treatment, additional research is needed on the efficacy of anticipatory temperament guidance in early childhood prevention programs.
